# Dietary inclusion of black soldier fly (*Hermetia illucens*) larvae meal, with exogenous protease supplementation, in practical diets for striped catfish (*Pangasius hypophthalmus*, Sauvage 1878)

**DOI:** 10.1371/journal.pone.0313960

**Published:** 2024-12-18

**Authors:** Rabia Haider, Noor Khan, Ayesha Aihetasham, Hafiz Abdullah Shakir, Mahroze Fatima, Ayesha Tanveer, Sheeza Bano, Wazir Ali, Maryam Tahir, Muhammad Asghar, Areej Farooq, Saba Aftab, Abrar Ul Haq, Mehwish Sarwar

**Affiliations:** 1 Institute of Zoology, University of the Punjab, Lahore, Pakistan; 2 Department of Fisheries and Aquaculture, University of Veterinary and Animal Sciences, Lahore, Pakistan; Tanta University Faculty of Agriculture, EGYPT

## Abstract

Fishmeal (FM) is a key component of commercial fish feeds, but due its unsustainable supply, the search for quality alternatives of FM has become a significant area of investigation worldwide. The insect-based proteins such as black soldier fly larvae (BSFL) are being recognized as an alternative ingredient. However, anti-nutritional factors in these alternatives may negatively affect nutrient utilization in fish. Incorporating exogenous protease enzymes as feed additives could be a promising way to improve the digestibility of these alternative ingredients. Therefore, this study aimed to evaluate the impact of dietary inclusion of BSFL meal, combined with protease supplementation, on various parameters of striped catfish (*Pangasius hypophthalmus*). Five diets were formulated: a control diet (FM-based), two diets with 30% and 60% BSFL inclusion (BSF30 and BSF60), and two diets with the same inclusion levels plus the incorporation of exogenous protease (BSF30P and BSF60P). One hundred fifty fish (8.26±0.02 g) were arbitrarily allocated to five groups, each with three replicates. The fish were provided with their respective diets twice per day for 60 days. In comparison to the control, fish-fed diets supplemented with protease demonstrated statistically significant lower levels of feed conversion ratio and higher growth performance regarding the final body weight and weight gain. Lipase activity in the BSF60P group was notably greater than in the control group. Protease supplementation significantly enhanced the apparent digestibility coefficient of protein, intestinal protease activity, and crude protein content in the whole body. Most hematological and biochemical parameters remained unaffected except for substantially lower triglyceride and cholesterol levels in the highest BSFL inclusion groups. These groups also showed a reduction in crude fat contents. While glutathione peroxidase and malondialdehyde levels did not change significantly from the control, the liver tissues of fish fed BSF60P diets showed considerably higher levels of antioxidant enzymes such as catalase and superoxide dismutase. The findings suggest that including 60% of a BSFL based diet, along with exogenous protease supplementation, is feasible without compromising the growth performance and health of striped catfish.

## 1. Introduction

Over the past few decades, there has been a notable surge in aquaculture growth and the global use of cultured fish [1]. The availability of conventional protein sources, specifically fish meal, for the formulation of aquatic feed has encountered significant deficits as a result of this expansion. The current use of fish meal from wild fisheries in aquafeed faces limitations due to stagnant catches, unpredictable availability caused by natural events, and increasing feed production [2, 3]. Therefore, finding sustainable and cost-effective substitutes has become a critical research direction in aquatic animal nutrition to ensure the sustainability of aquaculture [4].

Insects have been recognized as an eco-friendly, renewable, and sustainable alternative protein source for aquafeed production, with favorable quantity, quality, and acceptable nutritional composition [5, 6]. Among the various species evaluated to date, the black soldier fly (BSF, *Hermetia illucens* Linnaeus, 1758) has emerged as a viable option for sustainable large-scale production. This is attributed to its omnivorous dietary habits and its capacity to assimilate nutrients from a range of organic wastes and transform them into valuable, high quality proteins (protein>40%) and lipids (lipid>30%) suitable for animal consumption [7, 8]. The nutritional profile of BSFL varies depending on the organic waste used as a growing substrate [9, 10]. Compared to other insects, the essential amino acid profile of BSF larvae meal is more balanced and closely resembles fishmeal [6, 11, 12]. It also contains bioactive substances, including lauric acid, chitin, and antimicrobial peptides, which can have beneficial health effects in fish [13]. Moreover, unlike other fly species, *H*. *illucens* is not a disease vector [14].

Substituting FM with BSFL meal has shown mixed but promising results in various fish species [6, 15, 16]. Nonetheless, many studies suggest that BSF larvae meal should not account for more than 30% of the diet; otherwise, it may impair the growth performance and well-being of fish [5, 15, 17]. On the other hand, some research shows that a full replacement of FM with BSFL meal is possible [18, 19]. It is generally accepted that chitin in the insect exoskeletons, an unbranched polymer of N-acetylglucosamine, restricts the use of insect meals in aquafeeds [5].

To enhance the accessibility of nutrients and retention in fast-growing aquatic species, the post-harvest processing of BSFL proteins should be explored. Exogenous protease enzyme supplementation may be an effective strategy for improving nutrient digestion while using novel insect meals. Proteases are enzymes responsible for hydrolyzing complicated protein molecules into simpler amino acids [20]. Dietary supplementation of exogenous proteases can stimulate endogenous proteolytic enzyme activities, promoting nutrient assimilation and uptake in fish [21]. Although chitinase would ideally be employed to counteract the effects of chitin by breaking down the N-acetylglucoseamine polymer, the current lack of commercially available chitinase with sufficient activity and economic feasibility for aquaculture renders it impractical [20]. That is why a commercially available protease was included in a group of test diets to assess its effectiveness.

This approach has proven successful in replacing fishmeal with plant-based meals, resulting in improved growth performance and overall health in various fish species [22, 23]. Promising results have also been observed with protease supplementation in low fishmeal poultry by-product-based diets in rohu [24]. However, there are few reports of protease supplementation in insect-based diets for fish.

Striped catfish (*Pangasius hypophthalmus*, Sauvage 1878), which belongs to the Pangasiidae family, has been extensively farmed because of its large stocking density tolerance, rapid growth, and global demand. Nevertheless, high feed costs are a limiting factor in its profitability. Therefore, developing fishmeal-free, inexpensive feed could enhance production and profit margins. Although BSFL has been previously tested as an alternative protein source at different inclusion levels in fish diets, this research focuses on the synergistic effect of BSFL meal incorporation with exogenous protease supplementation, a relatively unexplored area in aquaculture. The present investigation aimed to understand the implications of exogenous protease supplementation on the utilization of nutrients as well as the impacts of using BSFL meal as an alternative for fishmeal in striped catfish diets. The primary research objectives were investigated via nutrient digestibility assessment, growth performance, whole-body proximate composition, digestive and antioxidant enzymes, as well as hematological and serum biochemical parameters of striped catfish.

## 2. Materials and methods

The current research study was performed at the Aquaculture and Fisheries Laboratory, Institute of Zoology, University of the Punjab, Lahore, Pakistan, after seeking approval from the ethical review board of the institute.

### 2.1 Diets preparation and experimental design

Feed materials were purchased from the regional market and suppliers. All the ingredients were dried in sunlight, crushed to a particle size of 0.05 mm, and then chemically analyzed for proximate evaluation, following the standard techniques by Association of Analytical Chemists (AOAC) [25]. Table 1 provides the proximate composition of both BSFL meal and fishmeal for comparison. A list of all the ingredients used in the test diets is given in Table 2. Exogenous protease (CIBENZA DP® 600,000 U/g) was added to the diet groups in the required amounts by dissolving it in 200 ml of water at 37°C. The solution was added before the feed dough was formed after being incubated for 24 hours at room temperature. Five diets were formulated, two with increasing percentages of BSFL meal at 30% (BSF30) and 60% (BSF60), and two additional diets with the same percentages of BSFL meal plus the incorporation of exogenous protease (BSF30P and BSF60P). A fifth diet, serving as a reference diet (Control), did not include BSFL meal or protease. The ground materials were thoroughly blended using an electric mixer, and feed dough was prepared by adding an adequate amount of distilled water. Using a hand pelletizer, 3 mm pellets were made from the dough. After complete drying, the test diets were kept in airtight polythene bags, labeled, and stored at -4°C until future use. Prior to the experiment, feed nutrition was analyzed using the official AOAC methods of analysis [25]. The feed nutrition analysis is presented in Table 2.

**Table 1:** Proximate analysis (%) of BSFL meal and fishmeal used in test diets.

Nutrient	BSFL meal	Fishmeal
Moisture	11.15	9.62
Crude Protein	47.81	54.30
Crude Fat	16.42	9.15
Ash	12.09	14.28

**Table 2:** Formulation and proximate composition of the experimental diets (% dry matter).

Ingredients	Experimental Diets				
	Control	BSF30	BSF60	BSF30P	BSF60P
Fishmeal^a^	35.00	24.50	14.00	24.50	14.00
Insect meal^b^	-	10.50	21.00	10.50	21.00
Soybean meal^a^	15.00	15.00	15.00	15.00	15.00
Corn meal^a^	8.00	8.00	8.00	8.00	8.00
Corn gluten^a^	14.00	14.00	14.00	14.00	14.00
Fish oil^a^	5.00	5.00	5.00	5.00	5.00
Wheat flour^c^	20.00	20.00	20.00	19.965	19.965
Vitamin premix^d^	1.00	1.00	1.00	1.00	1.00
Mineral mixture^e^	1.00	1.00	1.00	1.00	1.00
Protease (mg/kg)^f^	-	-	-	0.035	0.035
Cr_2_O_3_^f^	1.00	1.00	1.00	1.00	1.00
Total	100.00	100.00	100.00	100.00	100.00
***Nutrient composition (% dry matter)***					
Crude protein	45.89	45.60	45.16	45.20	45.01
Crude fibre	4.47	4.66	4.31	4.56	4.52
Crude fat	7.26	8.94	9.19	9.12	9.55
Ash	8.47	8.86	8.27	8.36	8.67

a Aqua feeds, Pvt Ltd., Multan, Pakistan

b Protein Worms House, Lahore, Pakistan

c Ravi Flour Mill, Lahore, Pakistan.

d Vitamin premix contained the following per kilogram; vitamin D3 480000 IU, 60000 mg inositol, 2400 mg vitamin E, 10 mg vitamin B12, vitamin A 10000 mg, 4000000 IU, 2400 mg vitamin K3, 4000 mg vitamin B1, 4000 vitamin B6, 1200 mg folic acid, 40000 mg vitamin C, 100 mg D-Biotin, 4000 mg Niacin, Cal. D. Pantothenate.

e Mineral mixture contained the following per kilogram; magnesium 200000 mg, selenium 100 mg, cobalt 2000 mg, manganese 23750 mg, iodine 2750 mg, zinc 75000 mg, copper 5000 mg.

f Ghazi Brothers, Karachi, Pakistan

### 2.2 Experimental fish and feeding management

Striped catfish juveniles with an average initial body weight of 8.18 ± 0.03 g/fish were obtained from Tawakkal Fish Hatchery in Muzaffargarh, Punjab, Pakistan. The juveniles were given a KMnO4 bath (5 g/L) for 1–2 minutes, followed by a two-week acclimation period in indoor fiberglass tanks, where they were provided with a control diet (5% of body weight/day). After acclimation, one hundred fifty juveniles (8.26±0.02, mean±SD) were arbitrarily divided into five groups (at a density of 10 fish/ tank), each with three replicates in 15 glass tanks (70×40×40 cm).The daily ration was offered twice a day (09:00 and 16:00), and all uneaten feed was removed daily by siphoning to determine feed intake (g) and calculate the feed conversion ratio. Key physicochemical parameters, including temperature, dissolved oxygen (DO), and pH (27.45 ± 0.3°C, 7.3 ± 0.1 mg/L, and 7.6 ± 0.1, respectively) were monitored daily to maintain optimal water quality for *P*. *hypophthalmus*.

### 2.3 Animal ethics

This study was conducted after seeking approval from the Ethical Review Board of University of the Punjab, Lahore, Pakistan (D/107/FIMS, 04-08-2023).

### 2.4 Sampling regime

After a 60 days trial period, the fish were weighed to assess their growth performance. The fish underwent a fasting duration of 24 hours. The fish in each tank were sedated using clove oil (6 ml/L) (Sigma Aldrich) and subsequently euthanized following blood sampling. Blood samples were drawn from the caudal vein of fish using a 1 ml syringe. Two tubes were used to collect blood samples: one containing 10% EDTA (etheylenediaminetetraacetic acid) as an anticoagulant for hematological analysis and the other without anticoagulant for biochemical assessment. To extract nonhemolysed serum, the anticoagulant-free samples were left to clot at room temperature for 30 minutes, after which they were centrifuged at 3000 rpm for 10 minutes. The resulting serum samples were collected and kept at -20°C until biochemical analysis. For the proximate analysis, whole fish from the pre-trial group (n = 3) and whole fish (n = 3/tank) at the conclusion of the experiment were randomly selected and frozen at -20°C for further analysis. The remaining fish were dissected to obtain liver and intestine samples for the evaluation of antioxidant and digestive enzyme activities, respectively.

### 2.5 Growth performance indices and morphometric calculations

After the 60-day feeding trial, the individual forktail length and body weight of the fish were measured, and the growth performance and morphometric indices were calculated using the following equations:

Weightgain(WG,g)=(Finalweight(g)−Initialweight(g)) (1)

$$Feed\;Intake\;(FI,\frac{g}{fish})=Feed\;given\;(g)-Unconsumed\;feed\;(g)$$ (2)

FeedConversionratio(FCR)=Totalfeedintake(g)/weightgain(g) (3)

$$Specific\;growth\;rate\;SGR\;(\%/day)=\frac{ln(average\;final\;weight)-ln(average\;initial\;weight)}{Experimental\;period\;(days)}\times 100$$ (4)

Survivalrate(%)=100×(Numberoffishattheendoftrial/Initialfishnumber) (5)

Hepatosomaticindex(HSI%)=100×weightoftheliver(g)/finalbodyweight(g) (6)

Viscerosomaticindex(VSI%)=100×weightoftheviscera(g)/finalbodyweight(g) (7)

Conditionfactor(CF)=bodyweight(g)/bodylength^3(cm)×100 (8)

### 2.6 Proximate composition analysis

Prior to proximate evaluation, the feed samples were pulverized using a laboratory mill. A representative sample was used to determine the moisture content of frozen whole-body samples that had been chopped and combined. The AOAC procedures [25] were followed to assess the proximate composition of BSFL meal, fish meal, test diets, and the whole body in triplicate. Analysis of moisture, total ash, crude protein, crude fat, and gross energy was performed as follows: a hot-air oven (Wise Ven) was used to measure the moisture content. It was set at 105°C for 12 hours until a steady weight was attained. A muffle furnace (Vulcan D-550) was used at 660°C for 5 hours to determine the ash contents. After the acid digestion, the crude protein was evaluated using a Kjeldahl protein auto-analyzer (Kjeltec^TM^ 8100) and a factor of 6.25 was applied to covert nitrogen in the crude protein. The crude fat was measured using a Soxhlet apparatus (Behro Test 901745) with diethyl ether extraction (40–60°C). Briefly, 1 g of sample was placed at Soxhlet thimble with defatted cotton wool on top, and 50–70 ml diethyl ether was added to the extraction cup. The solvent was then boiled for 15 min, the extraction knob was switched to the rising position for 20 minutes, and the residue was dried in an oven. After drying, the residue was placed in a desiccator for 5 min, weighed, and the crude fat content was calculated.

### 2.7 Evaluation of nutrient digestibility

A surrogate method was employed to measure the apparent digestibility coefficients (ADCs) of the experimental diets, using chromium oxide (Cr_2_O_3_) as an indigestible marker at a 1% inclusion level Table 2. Faecal sampling started a week after the test diets were fed to remove any recently ingested feed during the acclimation period. The tanks were cleaned daily with a 70% water exchange, to eliminate any leftover feed. Faecal matter was manually collected by siphoning and straining with a fine-meshed net before the morning meal. During the entire experimental period, the collected samples from each tank were combined into one sample and oven-dried for 6 hours at 50°C. The collected samples were subsequently analyzed for nutrients (proteins and lipids) following the methods outlined by AOAC [25]. The levels of Cr_2_O_3_ in the diets and faeces were estimated using the approach described by Furukawa and Tsukahara [26], which involved digesting the sample with concentrated nitric acid and oxidizing Cr_2_O_3_ with 70% perchloric acid. The absorbance value of the resultant solution was recorded at 350 mμ to calculate the Cr_2_O_3_ value using the given equation:

Chromiumoxide(%)=[{(absorbance−0.0032)/0.2089}/sampleweight]×100 (9)

The ADCs for dry matter, protein and lipids were calculated using the given equation [27, 28]:

Apparentdigestibilitycoefficient(%)=100−[100−(%chromiumoxideinfeed/%chromiumoxideinfaeces)×(%nutrientinfaeces/%nutrientindiet)] (10)

### 2.8 Digestive enzyme activities

Intestinal tissues from dissected fish (n = 4/replicate) were removed, pooled, and homogenized with a 0.25 M sucrose solution. The homogenized mixture was then centrifuged at 5000 rpm and 4°C for 15 minutes. The supernatant was stored at -20°C until the enzyme assay was performed. Protease activity was assessed using the casein digestion method of Kunitz [29]. One enzyme unit hydrolyzes casein at 37°C and pH 7.5, to produce a color equivalent to 1.0 μmole of tyrosine/minute. Amylase activity was measured using a 2% (w/v) starch solution as a substrate, following the dinitrosalicylic (DNS) acid method as outlined by Rick and Stegbauer [30]. Briefly, 0.5 mL starch and enzyme extract were incubated at 37°C for 30 min, followed by the cessation of the reaction with 1mL of DNS. The mixture was then boiled for 5 minutes, set to cool, diluted 5 times, and the absorbance was measured at 540 nm. Then, amylase activity was assessed by measuring the moles of maltose produced from starch per minute per milligram of protein at 37°C and using a maltose standard curve for calibration. Lastly, the lipase activity was measured spectrophotometrically using p-nitrophenyl palmitate (pNPP) as the substrate, following the method of Mahadik et al. [31].

### 2.9 Antioxidant enzyme activities

Liver samples (2g /replicate) were rinsed with phosphate buffer at pH 6.5 (0.2 M) to remove the RBCs, homogenized in cold buffer (1:4 w/v), and centrifuged for 15 minutes at 10,000 rpm and 4°C. The clear supernatants were then collected and refrigerated at -80°C until use in the enzyme assay. The catalase (CAT) activity was measured according to Chance and Mehaly’s method [32] by measuring the reduction of H_2_O_2_ concentration at a wavelength of 240 nm using a Thermo Scientific Evolution 60 UV-VIS spectrophotometer. The activity of glutathione peroxidase (GPx) was measured by determining its ability to reduce the concentration of H_2_O_2_ at 470 nm [33]. The superoxide dismutase (SOD) activity was assessed by determining its capacity to inhibit the photoreduction of nitroblue tetrazole (NBT) using the procedures outlined by Giannopolitis and Ries [34]. First, buffer, enzyme extract, and riboflavin were added to cuvettes and incubated for 12 minutes. Then, EDTA/NaCN and NBT were added before transferring the cuvettes to the spectrophotometer, followed by measuring the absorbance after 20 seconds of reaction to determine the SOD activity as percentage inhibition of NBT. Malondialdehyde (MDA) values were measured using the approach described by Gatta et al. [35]. Concisely, 1 g of liver samples from every specimen was homogenized in a Tris maleate and potassium chloride (KCl) solution and incubated at 37°C for 25 minutes after inserting ascorbic acid. Hydrochloric acid (HCl) and thiobarbituric acid (TBA) solution were added to the tubes, incubated at 95°C for 30 min, cooled, and then centrifuged after the addition of trichloroacetic acid (TCA). Ultimately, TBA values, expressed as 1g of MDA equivalents/mg of the sample, were measured photometrically at 530 nm.

### 2.10 Hematological and biochemical indices

Hematological parameters such as hemoglobin (Hb), red blood cells (RBCs), hematocrit (HCT), mean corpuscular volume (MCV), mean corpuscular hemoglobin (MCH), mean corpuscular hemoglobin concentration (MCHC), white blood cells (WBCs), and platelets (PLT) were examined using an automated hematological analyzer (Sysmex KX-21 N). The serum samples were used to determine glucose (GLU), total serum protein (TP), triglycerides (TG), cholesterol (CHOL), aspartate transaminase (AST), alkaline phosphatase (ALP), alanine transaminase (ALT), creatinine, and urea utilizing an automated biochemical analyzer (Hitachi 7600–110 Ltd., Japan).

### 2.11 Statistical analysis

All experimental data collected were statistically analyzed using IBM SPSS version 20. Any statistically significant (P<0.05) differences between the observed means were identified using analysis of variance (ANOVA). Multiple comparisons of means were also performed when ANOVA suggested significant variation between groups (P<0.05) using Duncan’s multiple range test (DMRT). The findings are expressed as the mean ± standard error (SE).

## 3. Results

### 3.1 Growth performance and feed utilization

All the experimental diets were readily accepted by the striped catfish. No mortality was recorded across all the groups. The results of growth performance, feed utilization, and biological indices are presented in Table 3. Dietary protease supplementation significantly increased the FBW and WG in striped catfish (P<0.05). The highest FBW and WG were observed in fish fed BSF30P diets, with values of 28.03±0.05 and 19.77±0.12, respectively. Furthermore, protease supplementation resulted in a statistically significant (P<0.05) effect on FCR in fish fed BSF30P diets (1.25±0.01) compared to the control and other treatment groups, though it is at a marginal level. However, no significant differences were observed in SGR, FI, HIS, VSI, and CF between the control and treatment groups (P>0.05).

**Table 3:** Growth, feed utilization and biological indices of *P*. *hypophthalmus* fed experimental diets.

Parameters	Treatments					P-value
	Control	BSF30	BSF60	BSF30P	BSF60P	
IBW (g fish^-1^)	8.18±0.03	8.26±0.02	8.21±0.10	8.25±0.01	8.26±0.03	*0*.*738*
FBW (g fish^-1^)	25.99±0.12^a^	25.94±0.13^a^	25.72±0.17^a^	28.03±0.05^b^	26.22±0.19^a^	*0*.*026*
WG (g fish^-1^)	17.74±0.12^a^	17.67±0.15^a^	17.51±0.13^a^	19.77±0.12^c^	18.05±0.13^b^	*<0*.*001*
FCR	1.27±0.003^b^	1.27±0.01^b^	1.28±0.003^b^	1.25±0.01^a^	1.27±0.006^b^	*0*.*037*
SGR (% day^-1^)	1.92±0.00	1.90±0.02	1.91±0.01	1.91±0.00	1.92±0.01	*0*.*485*
FI (g fish^-1^)	22.72±0.09	22.45±0.39	22.59±0.14	22.27±0.32	23.23±0.27	*0*.*205*
CF (K)	1.12±0.00	1.11±0.01	1.10±0.01	1.12±0.00	1.12±0.17	*0*.*181*
HSI (%)	1.22±0.05	1.21±0.01	1.21±0.005	1.22±0.005	1.21±0.07	*0*.*144*
VSI (%)	9.08±0.51	7.56±1.15	7.92±1.19	8.45±0.51	8.33±0.48	*0*.*424*

(Mean ± SE, n = 3)

Different superscripts in the same row represent a significant difference (P<0.05).

The survival rate was uniform across all groups.

IBW: initial body weight; FBW: final body weight; WG: weight gain; WG%: weight gain percentage; SGR: specific growth rate; FCR: feed conversion ratio; FI: feed intake; FE: feed efficiency; CF: condition factor; HSI: hepatosomatic index; VSI: Viscerosomatic index.

### 3.2 Whole body proximate composition

The crude protein and crude fat contents were significantly affected by the experimental diets (Table 4). The crude protein content significantly increased in the group fed diets supplemented with protease (i.e., BSF30P) and decreased in the groups with a higher concentration of BSFL meal (i.e., BSF60). Significant differences in crude fat content were recorded among the groups (P<0.05), with higher values in the BSF30 and BSF30P groups (4.12±0.11 and 4.02±0.04, respectively) and lower values in the BSF60 and BSF60P groups (3.38±0.08 and 3.14±0.07, respectively). However, the ash contents remained unaffected by the treatment.

**Table 4:** Whole body proximate of *P*. *hypophthalmus* fed experimental diets (% of dry weight).

	Treatments					P-value
	Control	BSF30	BSF60	BSF30P	BSF60P	
Dry matter	27.49±0.28^b^	26.72±0.23^b^	25.15±0.17^a^	28.74±0.35^c^	26.87±0.28^b^	*<0*.*001*
Crude protein	18.47±0.18^c^	17.33±0.11^b^	16.52±0.27^a^	18.38±0.23^c^	17.49±0.36^b^	*<0*.*001*
Crude fat	3.68±0.08^b^	4.12±0.11^c^	3.38±0.08^a^	4.02±0.04^c^	3.14±0.07^b^	*<0*.*001*
Ash	5.11±0.06	5.19±0.06	5.06±0.11	5.01±0.12	5.31±0.09	*0*.*190*

(Mean ± SE, n = 3)

Different superscripts in the same row represent a significant difference (P<0.05).

### 3.3 Antioxidant enzyme activity

The SOD and CAT enzyme activities of striped catfish were notably affected (P<0.05) by the test diets (Table 5). The highest SOD activity was observed in the BSF60P (1.23±0.02) and BSF30P (1.21±0.01) diets, while the lowest SOD activity was found in fish fed the control diet (1.11±0.00). CAT activities showed an increasing trend, with significantly higher and lower activity in fish fed BSF60P (14.13±0.16) and control diets (13.42±0.08), respectively. However, no notable differences were seen in GPx and MDA levels across the groups (Table 5).

**Table 5:** Antioxidant enzyme activity and MDA concentrations of *P*. *hypophthalmus* fed experimental diets.

Parameters	Treatments					P-value
	Control	BSF30	BSF60	BSF30P	BSF60P	
SOD (U/mg protein)	1.11±0.00^a^	1.13±0.00^a^	1.16±0.01^a^	1.21±0.01^b^	1.23±0.02^b^	*0*.*001*
CAT (U/mg protein)	13.42±0.08^a^	13.46±0.07^a^	13.56±0.07^ab^	13.88±0.11^bc^	14.13±0.16^c^	*0*.*003*
GPx (U/mg protein)	72.43±1.38	72.13±1.74	73.82±1.19	75.28±1.97	77.46±1.32	*0*.*206*
MDA (nmol/mg protein)	13.15±0.24^a^	13.27±0.1^b^	13.43±0.51^ab^	13.61±0.52^ab^	13.89±0.27^b^	*0*.*199*

(Mean ± SE, n = 3)

Different superscripts in the same row represent a significant difference (P<0.05).

SOD: superoxide dismutase; CAT: catalase; GPx: glutathione peroxidase; MDA: malondialdehyde.

### 3.4 Intestinal digestive enzyme activity

The experimental diets had a significant effect on the protease and lipase activities in the intestines of striped catfish (Table 6). Lipase activity was considerably elevated in the BSF60P group (707.49±5.28) in comparison to the control (692.67±2.89). Total protease activity also differed significantly among the experimental groups (P<0.05). The highest protease activities were shown by the BSF30P (4.29±0.01) and BSF60P (4.32±0.03) groups, while the BSF60 group had the lowest protease activity (4.19±0.02). There were no statistically significant variations in amylase activity across the treated groups (P>0.05).

**Table 6:** Digestive enzyme activity of *P*. *hypophthalmus* fed experimental diets.

Parameters	Treatments					P-value
	Control	BSF30	BSF60	BSF30P	BSF60P	
Amylase (U/g protein)	628.40±5.74	633.21±7.59	630.71±5.87	632.14±5.55	627.60±6.29	*0*.*590*
Protease (μmol/mg protein)	4.24±0.01^b^	4.23±0.01^b^	4.19±0.02^a^	4.29±0.01^c^	4.32±0.03^c^	*0*.*001*
Lipase (U/g protein)	692.67±2.89^a^	695.86±4.25^a^	704.25±4.88^b^	698.43±4.10^ab^	707.49±5.28^c^	*0*.*005*

(Mean ± SE, n = 3)

Different superscripts in the same row represent a significant difference (P<0.05).

### 3.5 Apparent digestibility coefficients (ADC_s_)

The ADCs of the fish fed test diets are given in Table 7. No significant variations were observed in the ADCs of dry matter and lipids (P>0.05). However, the ADC of protein considerably (P<0.05) increased in the diets supplemented with protease (BSF30P and BSF60P, 81.92±0.03 and 81.47±0.12, respectively), corresponding to that of the control (81.76±0.04), while the lowest ADC value of protein was recorded in the BSF60 group (77.31±0.14).

**Table 7:** Apparent digestibility coefficients (ADCs %) of *P*. *hypophthalmus* fed experimental diets.

Nutrient digestibility	Treatments					P-value
	Control	BSF30	BSF60	BSF30P	BSF60P	
Dry matter	75.42±0.17	75.38±0.01	75.30±0.07	75.41±0.02	75.46±0.02	*0*.*961*
Protein	81.76±0.04^d^	79.51±0.07^b^	77.31±0.14^a^	81.92±0.03^d^	81.47±0.12^c^	*<0*.*001*
Lipids	84.14±0.01^ab^	83.25±0.54^a^	84.38±0.15^b^	83.97±0.94^ab^	84.85±0.61^b^	*0*.*061*

(Mean ± SE, n = 3)

Different superscripts in the same row represent a significant difference (P<0.05).

### 3.6 Hematology and serum biochemistry

The experimental diets had no significant impact on serum biochemical parameters, including TP, GLU, urea, creatinine, and ALT, ALP, and AST activities, as shown in Table 8. However, the fish receiving BSF60 and BSF60P showed statistically significant lower levels of TG and cholesterol compared to both the control and the other test diets. No significant differences were found in RBC, Hb, Hct, WBC, MCH, MCV, MCHC, and platelet values (Table 9).

**Table 8:** Serum biochemical parameters of *P*. *hypophthalmus* fed experimental diets.

Parameters	Treatments					P-value
	Control	BSF30	BSF60	BSF30P	BSF60P	
TP (g/dL)	6.29±0.02	6.24±0.01	6.21±0.04	6.28±0.02	6.32±0.02	*0*.*076*
AST (U/mL)	13.13±0.34	13.18±0.31	14.22±0.58	13.43±0.21	14.15±0.32	*0*.*163*
ALP (U/L)	182.12±1.56	182.20±0.23	182.34±2.01	182.21±1.22	181.43±2.01	*0*.*268*
ALT (U/mL)	20.60±0.26	20.22±0.01	21.25±0.60	21.13±0.12	21.09±0.15	*0*.*177*
Creatinine (mg/dL)	0.59±0.21	0.56±0.24	0.97±0.04	0.44±0.07	0.54±0.23	*0*.*363*
Urea (mg/dL)	14.00±1.15	12.66±0.66	14.00±1.15	14.00±1.73	15.00±1.08	*0*.*852*
Chol (mg/dL)	160.73±2.38^b^	175.44±2.76^c^	146.74±3.23^a^	170.01±2.12^c^	152.15±3.49^ab^	*<0*.*001*
TG (mg/dL)	261.54±1.48^b^	266.09±2.78^bc^	231.08±2.29^a^	272.61±2.39^c^	226.02±1.79^a^	*<0*.*001*
GLU (mmol/L)	8.10±0.03	8.16±0.03	8.14±0.01	8.12±0.08	8.14±0.06	*0*.*614*

(Mean ± SE, n = 3)

Different superscripts in the same row represent a significant difference (P<0.05).

TP: total protein; AST: aspartate transaminase; ALP: alkaline phosphatase; ALT: alanine transaminase; Chol: cholesterol; TG: triglycerides, GLU: glucose.

**Table 9:** Hematological parameters of *P*. *hypophthalmus* fed experimental diets.

Parameters	Treatments					P-value
	Control	BSF30	BSF60	BSF30P	BSF60P	
WBCs (10^3^ ul^-1^)	14.30±0.56	14.20±0.30	14.26±0.78	14.10±0.40	14.40±0.15	*0*.*994*
RBCs (*10^6^ ul^-1^)	4.53±0.12	4.43±0.06	4.56±0.03	4.53±0.29	4.61±0.31	*0*.*976*
Hb (g/dl)	12.60±0.36	12.80±0.35	12.90±0.30	12.90±0.20	13.33±0.61	*0*.*756*
Hct (%)	36.66±0.88	35.66±0.66	36.33±0.88	37.66±0.33	37.00±0.57	*0*.*394*
MCV (fl)	76.04±0.05	76.04±0.01	75.41±0.60	75.74±0.57	76.09±0.06	*0*.*209*
MCH (pg)	27.00±0.57	27.00±0.57	27.33±0.88	27.66±0.33	28.00±0.57	*0*.*737*
MCHC (g/dl)	33.66±0.33	33.00±0.57	32.66±1.22	32.66±0.66	32.33±0.33	*0*.*722*
PLT (10^3^ ul^-1^)	171.33±3.88	172.00±3.57	174.33±5.66	173.33±3.96	170.66±4.20	*0*.*924*

(Mean ± SE, n = 3)

Different superscripts in the same row represent a significant difference (P<0.05).

WBC: white blood cells; RBC: red blood cells; Hb: hemoglobin; Hct: hematocrit; MCV: mean corpuscular volume; MCH: mean corpuscular hemoglobin; MCHC: mean corpuscular hemoglobin concentration; PLT: platelets.

## 4. Discussion

The quality of novel ingredients is crucial for achieving their optimal incorporation level into aquafeed formulations as replacements for fishmeal. This is essential for meeting the rigorous standards of the industry. Many freshwater fish species naturally consume insects as part of their diets. Therefore, adding insect meals to the feed can facilitate their consumption by fish [36]. Furthermore, dietary protease can improve protein breakdown and decrease the combustion of nitrogenous substances by metabolizing complex proteins into simpler amino acids [37]. Taking this into account, the current investigation sought to evaluate the effect of combining exogenous protease and insect protein, potentially to overcome the challenges posed by anti-nutritional factors, especially chitin in the insect exoskeleton.

Over the course of the 60-day feeding trial, all the treatment groups showed similar feed intake, indicating that the fish readily accepted the experimental diets. Additionally, the survival rate was also consistent across all treatments, suggesting that none of the diets had any adverse effects on the striped fish. However, the FBW and WG of the fish differed significantly among the treatments. The groups fed diets supplemented with exogenous protease (BSF30P, BSF60P) showed a notable enhancement in FBW and WG. The potential of protease to enhance nutrient bioavailability can be regarded as an explanation for this improvement, thus promoting growth performance and feed efficiency [38, 39]. Furthermore, because exogenous protease feeding activates endogenous enzymes, the fish might have easier access to amino acids [40]. The lower FCR in response to protease-supplemented diets can be linked to the same reason mentioned earlier. From the previous literature, it has been found that exogenous protease supplementation in low fishmeal diets enhanced the growth performance of various fish species [22, 41]. However, there are some studies that do not agree with this [42, 43]. In this study, no distinct variations were seen in terms of SGR and FI. These findings are supported by previous studies conducted on rainbow trout using BSFL-based diets supplemented with exogenous protease [20].

This study investigated the activity of digestive enzymes in the intestines of striped catfish. The results demonstrated no significant differences in amylase activity across the different treatments, suggesting that the test diets did not affect carbohydrate metabolism, which is consistent with findings from previous literature [44–46]. However, the protease activity was notably reduced in the BSF60 treatment (inclusion up to 210 g/kg). Similar decreases in protease activity were observed in meagre and snakehead when fed BSFL meal-based diets [47, 48]. This decrease can be attributed to the higher chitin content. Insect chitin is commonly recognized as a matrix composed of proteins, lipids, and other components. It has been proposed earlier that chitin in the diet might delay protease access in the gastrointestinal tract, thereby reducing intestinal protease activity [11]. The insoluble nature of chitin may impede water absorption from digesta, increase fecal volume, and prolong intestinal transit time, all of which could lower protease activity on substrates [49]. On the other hand, significantly higher protease activity was noted in the BSF30P and BSF60P treatments (HI inclusion up to 105 g/kg and 210 g/kg with protease supplementation). These results are in line with previous findings in *Labeo rohita* when low-fishmeal diets supplemented with protease were provided [24]. The lipase levels were likewise raised in these treatments. These results are consistent with prior research showing that dietary supplementation with protease can enhance the activity of endogenous enzymes in various fish species up to an optimal level [39, 50]. The activation of endogenous digestive enzymes may be the reason for the increase in activity of digestive enzymes observed in this study. According to Zhang et al. [51], a variety of factors, including the animal’s environment, diet, age, genetics, and state of health, can affect its ability to produce endogenous proteases in its gut.

Alterations in the dietary components can impact oxidative stress mechanisms in animals. Increased levels of reactive oxygen species (ROS) may cause DNA hydroxylation, protein denaturation, lipid peroxidation, and cellular damage [52]. In the current investigation, elevated levels of SOD and CAT were observed, indicating that the consumption of BSFL could potentially bolster the antioxidant defense system in juvenile striped catfish. This effect is likely attributable to the antioxidant properties of chitin found in insect exoskeletons [53]. Similar elevated levels of SOD and CAT were observed in the hepatic tissues of snakehead [48] and golden pompano [54] when fed BSFL based diets. Moreover, the same elevation of these enzymes was also found in the serum of snakehead [48], European seabass [55], African catfish [56], rohu, and *Catla catla* [57]. No discernible variations were observed in GPx and MDA content among the treatments. After being fed BSF larvae-based diets, comparable findings were also reported in snakehead [48], rainbow trout [58], meagre [59], African catfish [56], rohu, and *Catla catla* [57]. The consistent MDA levels observed in this study suggest that incorporating BSFL may not have influenced cellular polyunsaturated fatty acids, thereby potentially protecting striped catfish from oxidative stress.

In this investigation, the ash contents exhibited no significant variation across all feeding groups, which is comparable to previous studies on Nile tilapia [60] and African catfish [56]. As the inclusion of BSFL meal increased, there was a significant decrease in crude fat content. This aligns with earlier research by Kroeckel et al. [61], who observed a reduction in total lipid content in turbot with an increasing proportion of BSF prepupal meal in the diet. Similar trends were found in yellow catfish [62], Jian carp [44], and rice field eel [45]. Conversely, the crude protein content significantly increased in fish fed protease incorporated diets. This increase can be attributed to improved nutrient digestibility and feed utilization, as observed in our study with the incorporation of protease into the striped fish diet. These findings align with previous studies on African catfish [63] and rohu [24]. On the other hand, Yigit et al. [43] and Bolton et al. [20] found that CP contents in fish were unaffected by the dietary addition of protease. The variation observed in these outcomes could stem from differences among fish species, experimental conditions, dietary composition, and the levels of supplemented protease.

Hematological and biochemical analysis has proven to be an effective approach for measuring the impact of an experimental diet on the overall well-being and nutritional status of fish [64]. This research implies that dietary inclusion of BSF may not impact fish immune systems and hepatopancreatic health, as indicated by consistent serum AST and ALT levels, which aligns with previous research by Wang et al. [65]. The elevated levels of AST and ALT are indicative of hepatopancreatic damage [44]. The lower serum cholesterol and triglyceride levels observed in striped catfish after consuming the BSF60 and BSF60P diets correspond to findings from previous studies on *Pangasianodon hypopthalmus* [46], European seabass [49], and Jian carp [44]. These studies suggest that higher levels of BSF significantly reduced the triglyceride content, possibly due to the chitin in BSFL meal, as chitin has a high percentage of chitosan, which is believed to possess cholesterol-lowering properties in fish. According to reports, chitosan attaches to lipid (cholesterol) micelles, preventing cholesterol absorption and increasing bile acid excretion [66]. These results also highlight the beneficial role of protease in maintaining the normal activity of the cardiovascular system. Conversely, no comparable outcomes were observed for Russian sturgeon [67], Japanese seabass [68], or Atlantic salmon [18]. Similarly, Liu et al. [50] reported that protease addition had no considerable impact on serum lipid levels. This disparity may be linked to the distinct fish species and their dietary formulations. The current investigation reveals no significant hematological variations between the control and treatment groups, which is consistent with previous literature [18, 46, 56].

In the present study, ADC of protein values were lower in BSF60 (BSF inclusion up to 210 g/kg-without protease supplementation) and higher in BSF30P (BSF inclusion up to 105 g/kg-with protease supplementation) compared to the control diet (FM-based). Similar findings suggesting that dietary protease supplementation improves the digestibility of nutrients and growth performance have been reported for tilapia and rohu fed low-fishmeal diets by Li et al. [69] and Maryam et al. [24], respectively. Exogenous protease activates endogenous peptidase synthesis, enhancing protease activity and improving amino acid availability and nutrient digestibility [21]. On the contrary, no significant outcomes of protease supplementation were observed by Yigit et al. [43] and Huan et al. [70]. This discrepancy may be due to the fact that fish lacking a stomach maintain a neutral or alkaline environment in their digestive system. Given the low pH of stomach secretions, acidic proteases are required [71]. It has been reported that chitin negatively influences protein utilization, which could explain the low protein digestibility with increasing levels of dietary BSF meal incorporation [72, 73]. In this study, the ADC of dry matter and lipids showed no notable variation between the feeding groups. These results are comparable with past research on European seabass [49], meagre [47], and snakehead [48]. On the other hand, turbots given a diet containing 300 g/kg BSFL meal showed reduced lipid digestibility [61]. This may be explained by species-specific variations in addition to the higher proportion of BSFL meal lipids to the overall dietary lipid content in the turbot’s diet composition.

## 5. Conclusions

The findings of this investigation suggest that incorporating up to 60% of BSFL meal into the diets of striped catfish, along with protease supplementation, yields a viable protein and energy concentrate without any adverse effects on growth performance, proximate composition, lipid peroxidation, or nutrient digestibility. Further research on the synergistic effects of exogenous enzymes and insect proteins is still needed to fully comprehend their advantages and utility as supplements for other animal-based proteins.

## Supporting information

S1 File (XLSX)
